# Antibiotic resistance and molecular epidemiology of *Staphylococcus aureus *in Nigeria

**DOI:** 10.1186/1471-2180-11-92

**Published:** 2011-05-05

**Authors:** Adebayo O Shittu, Kenneth Okon, Solayide Adesida, Omotayo Oyedara, Wolfgang Witte, Birgit Strommenger, Franziska Layer, Ulrich Nübel

**Affiliations:** 1 Department of Microbiology, Obafemi Awolowo University, Ile-Ife, Nigeria; 2 Department of Medical Microbiology, University of Maiduguri Teaching Hospital, Maiduguri, Nigeria; 3 Molecular Biology and Biotechnology Division, Nigerian Institute of Medical Research, Lagos, Nigeria; 4 Department of Biological Sciences, College of Science, Engineering and Technology, Osun State University, Osogbo, Nigeria; 5 Robert Koch Institute, 38855 Wernigerode, Germany

## Abstract

**Background:**

*Staphylococcus aureus *is an important pathogen causing a wide range of infections in the hospital and community setting. In order to have adequate information for treatment of *S. aureus *infections, it is crucial to understand the trends in the antibiotic-resistance patterns. In addition, the occurrence and changes in types of *S. aureus*, clonal identities, and their geographic spread is essential for the establishment of adequate infection control programmes. In this study, 68 *S. aureus *isolates obtained from clinical and non-clinical sources in Nigeria between January and April 2009 were characterized using phenotypic and molecular methods.

**Results:**

All the *S. aureus *isolates were susceptible to teicoplanin, vancomycin, phosphomycin, fusidic acid, rifampicin, daptomycin, mupirocin, linezolid and tigecycline. Sixteen percent of the isolates were resistant to oxacillin, while 55% and 72% of isolates were resistant to tetracycline and trimethoprim/sulphamethoxazole (cotrimoxazole), respectively (Table 1). There was excellent correlation between the broth microdilution assay and detection of antibiotic resistance genes by the multiplex PCR, in the determination of *S. aureus *resistance to erythromycin, gentamicin, methicillin and tetracycline. A total of 28 *spa *types were identified in the study, and the predominant *spa *type among the methicillin-susceptible *S. aureus *(MSSA) isolates was t084 (13 isolates). The t037-ST241-SCC*mec*III type was the only clone identified in Maiduguri (North-East Nigeria) while in South-West Nigeria, diversity among the MRSA isolates (t451-ST8-SCC*mec*V; t008-ST94-SCC*mec*IV; t002-ST5-SCC*mec*V; t064-ST8-SCC*mec*V) was observed. The toxin genes *seh *and *etd *were detected in isolates affiliated with clonal complexes CC1, CC80 and sequence type ST25, respectively. The proportion of PVL-positive isolates among MSSA was high (40%). Most of the PVL-positive MSSA isolates were obtained from wound infections and associated with clonal complexes CC1, CC30, CC121 and with sequence type ST152.

**Conclusions:**

The use of phenotypic and molecular methods provided useful information on antibiotic resistance and molecular diversity of *S. aureus *in Nigeria. The high proportion of PVL-positive MSSA isolates affiliated to various clonal complexes and detected in all the health institutions is a major concern, both as a source of severe infections and as a potential reservoir that could lead to the emergence of PVL-positive MRSA. This study presents the first baseline information on the nature of the antibiotic resistance genes from *S. aureus *isolates in Nigeria. There is the need to curtail the spread and establishment of MRSA and PVL-positive MSSA clones in Nigerian health care institutions.

**Table 1:** Antibiotic resistance profile of *S. aureus *(MSSA and MRSA) from Nigeria

	**Number (%) of resistant isolates among**:		
Antibiotic	MSSA (n = 57)	MRSA (n = 11)	Total (n = 68)
Penicillin	49 (86)	11 (100)	60 (88.2)
Oxacillin	0 (0)	11 (100)	11 (16.2)
Teicoplanin	0 (0)	0 (0)	0 (0)
Vancomycin	0 (0)	0 (0)	0 (0)
Gentamicin	1 (1.8)	9 (81.8)	10 (14.7)
Tetracycline	27 (47.4)	11 (100)	38 (55.9)
Ciprofloxacin	12 (21.1)	8 (72.7)	20 (29.4)
Moxifloxacin	0 (0)	7 (63.6)	7 (10.3)
Trimethoprim/sulfamethoxazole	39 (68.4)	10 (90.9)	49 (72.1)
Phosphomycin	0 (0)	0 (0)	0 (0)
Fusidic acid	0 (0)	0 (0)	0 (0)
Erythromycin	2 (3.5)	6 (54.5)	8 (11.8)
Clindamycin	0 (0)	6 (54.5)	6 (8.8)
Rifampicin	0 (0)	0 (0)	0 (0)
Daptomycin	0 (0)	0 (0)	0 (0)
Mupirocin	0 (0)	0 (0)	0 (0)
Linezolid	0 (0)	0 (0)	0 (0)
Tigecycline	0 (0)	0 (0)	0 (0)

## Background

*Staphylococcus aureus *is a leading cause of diseases such as skin and soft tissue infections, pneumonia, bloodstream infections, osteomyelitis and endocarditis, as well as toxin-mediated syndromes like toxic shock and food poisoning [1,2]. It has developed resistance to a wide range of antimicrobial drugs, which complicates the treatment of infections. In particular, methicillin-resistant *S. aureus *(MRSA) has become a notorious etiologic agent for a wide variety of infections and it is one of the most important nosocomial pathogens worldwide [3-6]. Methicillin-susceptible *S. aureus *(MSSA) become MRSA through the acquisition and insertion into their genomes of a large DNA fragment known as staphylococcal chromosome cassette *mec *(SCC*mec*), which contains the methicillin resistance determinant, *mecA *[7]. Several variants of SCC*mec *have been described, which differ with respect to the composition of their recombinase genes and *mec *gene complex (containing the *mecA *gene) [8,9].

In the developing world, mortality associated with severe *S. aureus *infections far exceeds that in developed countries [10,11]. Recent studies have identified *S. aureus *as the main etiological agent of many infections in sub-Saharan Africa [12-16], and a number of investigations have reported that *S. aureus *is among the most frequently encountered bacterial species in microbiology laboratories in Nigeria [17-22]. However, data on the molecular epidemiology of this pathogen in Nigeria is very limited. Recent reports have indicated that the prevalence of hospital-associated MRSA varies in health care institutions [23,24]. A community-associated MRSA clone with a unique resistance profile has also been reported from South-West Nigeria [25]. To understand and potentially predict trends in antibiotic-resistance patterns and to establish adequate infection control programs, it is crucial to understand the local epidemiology of *S. aureus *in Nigeria. Knowledge of the local antimicrobial resistance patterns of bacterial pathogens is essential to guide empirical and pathogen specific therapy. The threat of antibiotic-resistant bacteria has initiated studies on the nature of genes encoding resistance and the mechanism by which these genes spread and evolve. Antibiotic susceptibility testing of *S. aureus *in Nigeria is based on phenotypic testing especially the disk diffusion technique but recent studies have relied on the PCR detection of the *mecA *gene for the identification and confirmation of MRSA [23-26]. However, no information is available on the nature of antibiotic resistance genes of *S. aureus *in Nigeria. Our present study provides baseline information on antibiotic resistance and molecular epidemiology of MSSA and MRSA in Nigeria.

## Results

### Antibiotic susceptibility testing and detection of antibiotic resistance genes in *S. aureus *isolates

The 68 *S. aureus *isolates obtained between January and April 2009 were analyzed for antimicrobial resistance (Table 1). All the isolates were susceptible to teicoplanin, vancomycin, phosphomycin, fusidic acid, rifampicin, daptomycin, mupirocin, linezolid and tigecycline, and two isolates were susceptible to all the antibiotics tested. In addition to the antibiotics stated above, all MSSA isolates (84%) were susceptible to clindamycin and moxifloxacin and less than 4% were resistant to erythromycin, 21.1% to ciprofloxacin, 47% to tetracycline, 68% to cotrimoxazole and 86% to penicillin. The predominant antibiotypes among the MSSA isolates were resistance to penicillin, tetracycline and cotrimoxazole (15 isolates), and resistance to penicillin and cotrimoxazole (13 isolates). A total of 11 isolates were resistant to oxacillin and confirmed as MRSA based on the detection of the *mecA *gene (Table 1). The *ermA *gene was identified in all erythromycin-resistant MRSA isolates, while two erythromycin-resistant MSSA isolates possessed the *msrA *gene. All the gentamicin-resistant isolates carried the *aacA-aphD *gene. Moreover, the *tetM *gene was detected in 11 isolates (7 MRSA and 4 MSSA) and the *tetK *gene was present in 4 MRSA and 23 MSSA isolates.

### SCC*mec *typing

The SCC*mec *type V was identified in four MRSA isolates obtained in Ile-Ife, Ibadan and Lagos, while one MRSA isolate from Ile-Ife possessed the SCC*mec *type IV element (Table 2). The MRSA isolates from Maiduguri were non-typeable for the SCC*mec *element based on established protocols [9,27], and no amplification was observed for the *ccrA*, *ccrB*, and *ccrh *genes. However, these MRSA isolates possessed the *ccu *gene. The comparison and analysis of the *ccu *sequences from two selected MRSA isolates in this group with sequences in the GenBank suggested that the MRSA isolates possessed an SCC*mec *type III element of uncommon organization, which had not been identified using standard protocols.

**Table 2:** Characterization of MRSA isolates from Nigeria based on antibiotic susceptibility pattern, detection of antibiotic resistance genes, SCC*mec *typing, *spa *typing and MLST

Isolate No	Location	Sample or Clinical Diagnosis	Antibiotype	Antibiotic resistance genes	SCC*mec *type	*spa *type	MLST
09-01730	Ile-Ife	Chronic ulcer	PEN, OXA, GEN, OTE, SXT	*mecA*, *aacA-aphD*, *tetK*	V	t451	8
09-01731	Ile-Ife	Urinary tract infection	PEN, OXA, GEN, OTE	*mecA*, *aacA-aphD*, *tetM*	IV	t008	94 (8-slv)
09-01739	Lagos	Wound infection	PEN, OXA, OTE, CIP, SXT	*mecA*, *tetK*	V	t002	5
09-01776	Ibadan	Conjunctivitis	PEN, OXA, OTE, SXT	*mecA*, *tetK*	V	t064	8
09-01786	Ibadan	Wound infection	PEN, OXA, GEN, OTE, CIP, SXT, MFL	*mecA*, *aacA-aphD*, *tetK*	V	t064	8
09-01789	Maiduguri	Wound infection	PEN, OXA, GEN, ERY, CLI, OTE, CIP, SXT, MFL	*mecA*, *aacA-aphD*, *ermA*, *tetM*	III	t037	241
09-01791-1	Maiduguri	Semen (Infertility)	PEN, OXA, GEN, ERY, CLI, OTE, CIP, SXT, MFL	*mecA*, *aacA-aphD*, *ermA*, *tetM*	III^a^	t037	241
09-01795	Maiduguri	Throat Infection	PEN, OXA, GEN, ERY, CLI, OTE, CIP, SXT, MFL	*mecA*, *aacA-aphD*, *ermA*, *tetM*	III^a^	t037	ND
09-01809	Maiduguri	Semen (Infertility)	PEN, OXA, GEN, ERY, CLI, OTE, CIP, SXT, MFL	*mecA*, *aacA-aphD*, *ermA*, *tetM*	III^a^	t037	241
09-01811	Maiduguri	Wound Infection	PEN, OXA, GEN, ERY, CLI, OTE, CIP, SXT, MFL	*mecA*, *aacA-aphD*, *ermA*, *tetM*	III^a^	t037	ND
09-01812	Maiduguri	Wound Infection	PEN, OXA, GEN, ERY, CLI, OTE, CIP, SXT, MFL	*mecA*, *aacA-aphD*, *ermA*, *tetM*	III^a^	t037	ND

^a^SCC*mec *type inferred from related isolate 09-01789

slv: single locus variant

Geographical region: South-West Nigeria (Ile-Ife, Ibadan and Lagos)

North-East Nigeria (Maiduguri)

KEY

PEN: Penicillin G; OX: Oxacillin; GEN: Gentamicin; ERY: Erythromycin; CLI: Clindamycin; OTE: Tetracycline; CIP: Ciprofloxacin; SXT: Trimethoprim/sulfamethoxazole; MFL: Moxifloxacin

ND: Not determined

### Molecular diversity of *S. aureus *based on *spa *typing and MLST

Twenty-eight *spa *types were identified in this study. Representative isolates were subsequently selected for MLST (Tables 2 and 3). Results indicated that nine major clonal complexes were represented in our strain collection from Nigeria (Tables 2 and 3). These clonal complexes plus one that we did not find (CC22) seem to predominate the *S. aureus *population on all continents. In addition, we found sequence type ST152, which has been reported previously in Ibadan and Maiduguri (Nigeria) [24,25].

### Detection of markers frequently associated with community-acquired *S. aureus*

A total of 23 of the 57 (40.3%) MSSA isolates (grouped in clonal complexes - CC1, CC5, CC15, CC30, CC121, CC80 and sequence type ST152) were PVL positive (Table 3), while none of the MRSA possessed the PVL gene. The enterotoxin H gene (*seh*) was detected in the isolates from clonal lineage CC1. Three MSSA isolates (ST25) from nasal samples of healthy individuals and one MSSA (CC80) from a wound infection possessed the *etd *gene. All the *S. aureus *isolates were *arcA *negative.

**Table 3:** Characterization of MSSA isolates from Nigeria by antibiotic susceptibility pattern, detection of antibiotic and virulence genes, *spa *typing and MLST

Isolate No	Location	Sample Or Clinical Diagnosis	Antibiotype	Antibiotic resistance genes	Toxin genes	*spa *type	MLST	Clonal Complex (CC)
09-01760	Ife	Wound Infection	PEN, CIP, SXT	-	*lukPV, seh*	t127		CC1
09-01823	Ife	Wound Infection	PEN, OTE	*tetK*	*lukPV, seh*	t127		
09-01785-1	Ibadan	Conjunctivitis	PEN	-	*lukPV, seh*	t114		
09-01787	Maiduguri	Wound Infection	PEN, OTE	*tetK*	*seh*	t321	1	

09-01733	Ife	Otitis media	PEN, SXT	-	*lukPV*	t311		CC5
09-01738	Lagos	Urinary tract infection	PEN, ERY, OTE, CIP, SXT	*tetM, mrsA*	-	t311	5	
09-01777	Ibadan	Wound Infection	PEN, CIP	-	-	t311		
09-01815	Maiduguri	Otitis media	PEN, ERY, OTE, SXT	*tetM, mrsA*	-	t311		

09-01737	Lagos	Semen (Infertility)	PEN, GEN, OTE, SXT	*aacA-aphD, tetK*	-	t951		CC8
09-01742	Lagos	Unknown	PEN, OTE	*tetK*	-	t1617	8	
09-01780	Ibadan	Conjunctivitis	PEN, SXT	-	-	t064		
09-01796	Maiduguri	Sputum (Unknown)	PEN, OTE, SXT	*tetK*	-	t064		
09-01810	Ife	Sputum (Unknown)	PEN, OTE, CIP, SXT	*tetK*	-	t1496		
09-01817-1	Maiduguri	Urinary tract infection	PEN, OTE, SXT	*tetK*	-	t1496		
09-01819	Maiduguri	Semen (Infertility)	PEN, OTE, SXT	*tetK*	-	t2812		
09-01822	Ife	Sputum (Unknown)	PEN, OTE, SXT	*tetK*	-	t1496		

09-01734	Ife	Unknown	PEN, OTE, SXT	*tetK*	-	t084		CC15
09-01736	Lagos	Otitis media	OTE	*tetM*	-	t084		
09-01750	Ife	Nasal*	PEN, SXT	-	*lukPV*	t084		
09-01751	Ife	Nasal*	PEN, SXT	-	*lukPV*	t084	15-slv	
09-01752	Ife	Nasal*	SXT	-	*lukPV*	t084		
09-01755	Ife	Nasal*	PEN, OTE, SXT	*tetK*	-	t084		
09-01756	Ife	Nasal*	PEN, OTE, CIP, SXT	*tetK*	-	t084		
09-01799	Maiduguri	Otitis media	PEN, OTE, SXT	*tetK*	-	t084		
09-01801	Maiduguri	Otitis media	PEN, OTE, SXT	*tetK*	-	t084		
09-01805	Ife	Blood Infection	OTE	*tetM*	-	t084	15-slv	
09-01820	Ife	Wound Infection	PEN, SXT	-	-	t084		
09-01821	Ife	Wound Infection	PEN, OTE, SXT	*tetK*	-	t084		
09-01824	Ife	Wound Infection	CIP, SXT	-	-	t084		
09-01788	Maiduguri	Wound Infection	PEN, OTE, SXT	*tetK*	-	t2216		
09-01798	Maiduguri	Semen (Infertility)	PEN, OTE, SXT	*tetK*	-	t2216	15	
09-01804	Maiduguri	Wound Infection	PEN, OTE, SXT	*tetK*	-	t2216		
09-01806	Ife	Unknown	PEN, SXT	-	-	t328		
09-01813	Ife	Wound Infection	PEN, SXT	-	-	t5387		

09-01740	Lagos	Wound Infection	PEN, CIP, SXT	-	*lukPV*	t318		CC30
09-01743	Lagos	Wound Infection	CIP, SXT	-	*lukPV*	t318	30	
09-01747-2	Lagos	Wound Infection	Susceptible to all antibiotics	-	*lukPV*	t318	30	
09-01779	Ibadan	Wound Infection	PEN, CIP, SXT	-	*lukPV*	t021		
09-01825	Ife	Otitis media	PEN, CIP	-	-	t631		
09-01732	Ile-Ife	Unknown	PEN, SXT	-	*lukPV*	t159		CC121
09-01759	Ile-Ife	Wound Infection	PEN, SXT	-	*lukPV*	t314		
09-01797	Maiduguri	Wound Infection	OTE	*tetK*	*lukPV*	t314		
09-01826	Ile-Ife	Otitis media	PEN	-	*lukPV*	t159	121	
09-01745	Lagos	Semen (Infertility)	PEN, SXT	-	*lukPV*	t2304	121	
09-01781	Ibadan	Wound Infection	PEN, OTE, SXT	*tetK*	*lukPV*	t2304		

09-01761	Ife	Wound Infection	PEN	-	*lukPV*	t355		singleton
09-01762	Ife	Wound Infection	PEN	-	*lukPV*	t355		
09-01778	Ibadan	Wound Infection	PEN, CIP	-	*lukPV*	t355		
09-01790	Maiduguri	Wound Infection	Susceptible to all antibiotics	-	-	t355	152	
09-01793	Maiduguri	Wound Infection	PEN	-	*lukPV*	t355		
09-01803	Maiduguri	Wound Infection	PEN, OTE, SXT	*tetK*	*lukPV*	t355		

09-01753	Ife	Nasal*	PEN, SXT	-	*etd*	t3772	25	singleton
09-01754	Ife	Nasal*	PEN, SXT	-	*etd*	t3772		
09-01757	Ife	Nasal*	PEN, SXT	-	*etd*	t3772		

09-01802	Maiduguri	Wound Infection	PEN, CIP	-	-	t939	45	CC45

09-01792	Maiduguri	Wound Infection	PEN, OTE	*tetK*	-	t458	97	CC97

09-01800	Maiduguri	Wound Infection	PEN, OTE, SXT	*tetK*	*lukPV, etd*	t934	80	CC80

^a^Clonal complex (CC) inferred from MLST and *spa *typing

*Nasal isolates from apparently healthy male students

slv: single locus variant

Geographical region: South-West Nigeria (Ile-Ife, Ibadan and Lagos)

North-East Nigeria (Maiduguri)

## Discussion

There was excellent correlation between the broth microdilution method and detection of the genetic determinants by multiplex PCR for *S. aureus *resistance to erythromycin, gentamicin, methicillin and tetracycline (Tables 2 and 3). About 55% (11 MRSA, 27 MSSA) and 70% (10 MRSA, 39 MSSA) of the *S. aureus *isolates were resistant to tetracycline and cotrimoxazole, and as previous studies from South-West Nigeria had shown [23,25], it appears that there is a high proportion of *S. aureus *isolates resistant to these antibiotics in Nigeria. Tetracycline and cotrimoxazole historically had wide clinical application, is inexpensive, orally administered and available from diverse sources where they are sold with or without prescription in Nigeria. Moreover, they are listed in many developing countries as among the antibacterial agents that have been rendered ineffective, or for which there are serious concerns regarding bacterial resistance [28]. It appears that misuse and overuse of these antibiotics could have contributed to this trend in Nigeria. Therefore, to prevent treatment failures in the absence of data on antibiotic susceptibility testing, public enlightenment on the ineffectiveness of these antibiotics against *S. aureus *infections, and the enactment of effective drug policies in Nigeria are urgently needed. The predominant mechanism of trimethoprim resistance in *S. aureus *appears to be mutation of the dihydrofolate reductase (DHFR), which is selected even when trimethoprim is used in combination with sulfamethoxazole [29]. In this study, all the trimethoprim-resistant *S. aureus *isolates were *dfrA *negative suggesting that mutation of the dihydrofolate reductase (DHFR) is responsible for resistance. Isolates resistant to tetracycline carried either one of the resistance genes *tetK *or *tetM *(Tables 2 and 3), which mediate resistance through active drug efflux or ribosomoal protection mechanisms, respectively. This is the first study that provides baseline information on the nature of the antibiotic resistance genes from *S. aureus *isolates in Nigeria. The multiplex PCR assay was easy to perform, cost-effective and assisted in the prompt characterization of the resistance genes stated above. It could be adapted for use by clinical scientists in the referral health care institutions regarding the antibiotics administered and the prevalent resistance determinants in Nigeria.

The proportion of PVL-positive isolates among MSSA was high (40%). Most of the PVL-positive MSSA isolates were obtained from wound infections and classified in clonal complexes CC1, CC30, CC121 and ST152. Moreover, the detection of the *seh *gene in CC1 isolates and the identification of the *etd *gene in ST25 and CC80 isolates is in agreement with previous reports [27,30-32]. PVL is frequently associated with severe and recurrent skin and soft-tissue infections (SSTIs) and has previously been found in *S. aureus *isolates from various complexes. In particular, PVL-producing MSSA affiliated to CC121 are known to be common in many countries on all continents [30,33,34], including Nigeria, Togo and South Africa in sub-Saharan Africa [25,30,35]. PVL-positive ST152 was the predominant clone in a study recently conducted in North-Eastern Nigeria [24] and it was the second most prevalent clone in a carriage study from a West-African country (Mali) [36]. Furthermore, the high prevalence of PVL positive MSSA ST152 emerging in the community as well as in hospitals in West Africa has also been described [31]. Hence, ST152 seems to be widespread and frequent in West Africa, whereas it is comparatively rare elsewhere [33,37], in contrast to many other clonal complexes that display worldwide occurrence. The *luk-PV *genes are carried on mobile genetic elements (prophages), which may be incorporated into *S. aureus *lineages through horizontal transfer, either before or after acquisition of the *mecA *gene [38]. The high proportion of PVL-positive MSSA observed in this study indicate that conditions that increase the risk of inter-individual transmission (e.g skin-to-skin and skin-to-fomite contacts) could represent important routes of spread in the various hospital settings. Contact with colonized and/or infected individuals as well as contaminated fomites in the spread of PVL positive *S. aureus *have been described as risk factors for community-associated MRSA [39]. Moreover, the detection of PVL-positive MSSA ST152 from members of one family and their relatives with skin infections at the Canary Island underscore the pathogenic and contagious nature of this clone [40]. More detailed investigations on the prevalence of PVL-positive *S. aureus *are needed in Africa with respect to (i) nasal carriage of *S. aureus *in the hospitals and community, (ii) cross-transmission from post-operative wound infections acquired during hospital stay, and (iii) cross-transmission from patients admitted to the health institutions for treatment of an SSTI acquired in the community. The detection of PVL-positive MSSA isolates from the various health institutions, indicating their wide geographical distribution, could pose serious problem in the future as potential reservoirs for resistance and virulence factors, and could lead to the emergence and spread of PVL-positive MRSA clones in Nigeria causing severe infections. This could have important implications for the enactment of effective infection control guidelines.

MRSA has become a major public health problem worldwide and recent reports have indicated that the prevalence of hospital-associated MRSA (based on the detection of the *mecA *gene) in health care institutions in Nigeria may vary from 1.5% to 20% [23-25]. All the MRSA isolates obtained from Maiduguri (North-East Nigeria) had the same *spa *type (t037) and MLST profile (ST241), identical to isolates from the same region that had been investigated in a previous study [24]. Another study [25] also reported that the clone was identified in a hospital in Ibadan (South-Western Nigeria). ST241 is a single locus variant (slv) of the ST239 clone, which is prevalent in South East Asia and has also been reported from Europe, the Americas [41], and several countries in Africa [6,42-44]. The multi-resistant nature of this MRSA clone could be explained by the presence of several resistance genes in the SCC*mec *cassette and it was recently shown to have spread across several continents since the 1960s [41]. MRSA ST239 demonstrating low level resistance to glycopeptides have been reported recently in Russia [45] and New Zealand [46]. In contrast, in South-Western Nigeria, we identified more diversity among the MRSA isolates. In three different hospitals in this region, we observed several different clones of MRSA that can be distinguished on the basis of MLST, SCC*mec *typing and *spa *typing, and displayed distinct antimicrobial resistance profiles (Table 2).

## Conclusions

This study showed that the combination of susceptibility testing and various molecular methods provided useful information on the antibiotic resistance and molecular diversity of *S. aureus *in Nigeria. Although the number of *S. aureus *isolates available for our investigation and epidemiological information was limited, the high proportion of PVL-positive MSSA observed in this study indicate that adequate measures are needed to curtail the spread and establishment of MRSA and PVL-positive MSSA clones in Nigerian health care institutions.

## Methods

### Isolation and identification of *S. aureus *isolates

In this study, a total of 68 non-duplicate consecutive *S. aureus *isolates (60 - clinical isolates; 8 - nasal isolates; one isolate per sample per individual) obtained between January and April 2009 were characterized. The clinical isolates were obtained from samples processed in the microbiology laboratories of referral health care institutions in Ile-Ife, Ibadan and Lagos (South-West Nigeria), and Maiduguri (North-East Nigeria), each of which are 500-bed facilities providing medical care to about one million people. The clinical isolates were cultured from 30 males (median age: 31 years, range: 1 year-70 years), 21 females (median age: 36 years, range: 1 week-63 years) and 9 unknown gender. In addition, nasal isolates were obtained from apparently healthy male undergraduate students in Ile-Ife. The origin and characteristics of each isolate is stated in Tables 2 and 3. The isolates were cultured on sheep blood agar and phenotypic identification of *S. aureus *was based on colony morphology and positive plasma coagulase reaction (slide and tube test). The susceptibility testing of the isolates to 18 antibiotics was performed using the broth microdilution assay as described by Deutsches Institut für Normung [47]. The antibiotic panel included penicillin G, oxacillin, teicoplanin, vancomycin, gentamicin, tetracycline, ciprofloxacin, moxifloxacin, trimethoprim/sulfamethoxazole (cotrimoxazole), phosphomycin, fusidic acid, erythromycin, clindamycin, rifampicin, daptomycin, mupirocin, linezolid and tigecycline.

### DNA extraction

Genomic DNA was obtained from a 2 ml overnight culture using a DNeasy tissue kit (Qiagen, Hilden, Germany) with lysostaphin (100 μg/ml) to achieve bacterial lysis.

### PCR detection of the *tuf *gene

Phenotypic identification of the *S. aureus *isolates was confirmed by the detection of the *tuf *gene [48].

### Multiplex PCR for detection of antibiotic resistance genes

The antibiotic resistance determinants investigated were the *aac-aphD *(aminoglycoside resistance) *mecA *(methicillin resistance) *ermA*, *ermC *(erythromycin resistance) and *tetK, tetM *(tetracycline resistance) genes. PCR primers and conditions were as described in a previously established protocol [49]. Moreover, the detection of the *dfrA *and *msrA *genes (trimethoprim resistance and macrolide efflux resistance determinants) were investigated using the following primers *tmp*I: CTC ACG ATA AAC AAA GAG TCA; *tmp *II: CAA TCA TTG CTT CGT ATA ACG and *msrA *f: GAA GCA CTT GAG CGT TCT; *msrA *r: CCT TGT ATC GTG TGA TGT which amplified a 201bp and 287bp of the *dfr *and *msrA *genes, respectively. The PCR conditions were as follows: Initial denaturation at 95°C for 2 minutes followed by 30 cycles of amplification with 94°C for 30 seconds, annealing at 50°C for 30 seconds, extension at 72°C for 30 seconds and final extension at 72°C for 4 minutes.

### Multiplex PCR for detection of markers associated with community-acquired *S. aureus*

A multiplex PCR reaction protocol [27] was used to detect markers associated with community-acquired *S. aureus*. They included the enterotoxin H gene (*seh*) for community-acquired *S. aureus *of clonal lineage ST1/USA400, the arginine deiminase gene (*arcA*) as part of the ACME (arginine catabolic mobile element) cluster for ST8/t008/USA300, the gene for exfoliative toxin D (*etd*) for ST80, and the Panton-Valentine Leukocidin (PVL) gene.

### SCC*mec *typing

SCC*mec *elements were classified by the multiplex PCR strategy [9,50]. SCC*mec *elements that could not be typed were characterized based on PCR amplification and sequence analysis of the cassette chromosome recombinases A and B genes (*ccrA*, *ccrB*), cassette chromosome helicase (*cch*) and another gene of unknown function (*ccu*) [51].

### *Spa *typing

*Spa *typing was based on the method described previously [52]. The nucleotide sequences were analyzed using the RIDOM Staph-Type software (Ridom GmbH, Germany) to assign the isolates to the various *spa *types.

### Multilocus sequence typing (MLST)

MLST was performed according to the previously published protocol [53].

## Authors' contributions

AOS, WW, BS, FL and UN conceived the study. KO, SA and OO participated in the preliminary identification of the isolates, AOS carried out the phenotypic and molecular characterization of the isolates. All authors read and approved the final version of the manuscript.
